# The Interactive Care Coordination and Navigation mHealth Intervention for People Experiencing Homelessness: Cost Analysis, Exploratory Financial Cost-Benefit Analysis, and Budget Impact Analysis

**DOI:** 10.2196/64973

**Published:** 2025-03-18

**Authors:** Hannah P McCullough, Leticia R Moczygemba, Anton L V Avanceña, James O Baffoe

**Affiliations:** 1 Health Outcomes Division, College of Pharmacy, The University of Texas at Austin, 2409 University Avenue, Austin, TX, 78712, United States, 1 512-232-6880; 2 Department of Internal Medicine, Dell Medical School, The University of Texas at Austin, Austin, TX, United States

**Keywords:** people experiencing homelessness, budget impact analysis, financial cost-benefit analysis, mHealth, care coordination, care, mobile health, smartphones, homeless, hospitalization, cost analysis, health care cost, economic, emergency department, United States, cost-benefit, digital health

## Abstract

**Background:**

The Interactive Care Coordination and Navigation (iCAN) mobile health intervention aims to improve care coordination and reduce hospital and emergency department visits among people experiencing homelessness.

**Objective:**

This study aimed to conduct a three-part economic evaluation of iCAN, including a (1) cost analysis, (2) exploratory financial cost-benefit analysis, and (3) budget impact analysis (BIA).

**Methods:**

We collected cost and expenditure data from a randomized controlled trial of iCAN to conduct a cost analysis and exploratory financial cost-benefit analysis. Costs were classified as startup and recurring costs for participants and the program. Startup costs included participant supplies for each participant and SMS implementation costs. Recurring costs included the cost of recurring services, SMS text messaging platform maintenance, health information access fees, and personnel salaries. Using the per participant per year (PPPY) costs of iCAN, the minimum savings reduction in the average health care costs among people experiencing homelessness that would lead to a benefit-cost ratio >1 for iCAN was calculated. This savings threshold was calculated by dividing the PPPY cost of iCAN by the average health care costs among people experiencing homelessness multiplied by 100%. The benefit-cost ratio of iCAN was calculated under different savings thresholds from 0% (no savings) to 50%. Costs were calculated PPPY under different scenarios, and the results were used as inputs in a BIA. A probabilistic sensitivity analysis was conducted to incorporate uncertainty around cost estimates. Costs are in 2022 US $.

**Results:**

The total cost of iCAN was US $2865 PPPY, which was made up of US $265 in startup (9%) and US $2600 (91%) in recurring costs PPPY. The minimum savings threshold that would cause iCAN to have a positive return on investment is 7.8%. This means that if average health care costs (US $36,917) among people experiencing homelessness were reduced by more than 7.8% through iCAN, the financial benefits would outweigh the costs of the intervention. When health care costs are reduced by 25% ($9229/$36,917; equal to 56% [$9229/$16,609] of the average cost of an inpatient visit), the benefit-cost ratio is 3.22, which means that iCAN produces US $2.22 in health care savings per US $1 spent. The BIA estimated that implementing iCAN for 10,250 people experiencing homelessness over 5 years would have a financial cost of US $28.7 million, which could be reduced to US $2.2 million if at least 8% ($2880/$36,917) of average health care costs among people experiencing homelessness are reduced through the intervention.

**Conclusions:**

If average costs of emergency department and hospital visits among people experiencing homelessness were reduced by more than 7.8% ($2880/$36,917) through iCAN, the financial benefits would outweigh the costs of the intervention. As the savings threshold increases, it results in a higher benefit-cost ratio.

## Introduction

People experiencing homelessness have higher rates of emergency department (ED) and hospital visits than housed groups. A systematic review reported that, on average, across 10 studies, the number of ED visits among people experiencing homelessness ranged from 0.7 to 5.8 visits annually. Furthermore, 5 of these studies compared ED use between people experiencing homelessness and housed groups and found that the rates were higher in people experiencing homelessness [1]. It has also been reported that people experiencing homelessness have 1.9 times the odds of hospital readmissions compared with housed groups [2]. Furthermore, a study of the hospital use patterns of people experiencing homelessness using electronic health data in an integrated health system in the United States found that people experiencing homelessness have higher rates of 30-day readmissions and longer lengths of stay in the hospital compared with those that are not homeless [3]. Some of the reasons for high ED and hospital utilization in people experiencing homelessness includes inadequate access to primary care and challenges taking medications due to medications being lost or stolen and competing priorities for basic needs such as shelter and food [4,5].

Mobile health (mHealth) is a type of digital technology that involves the use of mobile devices such as mobile phones to deliver health services [6]. The ubiquity and declining costs of mobile phones in the United States and other countries has generated interest in the use of mHealth to improve population and public health [7,8]. Studies have shown that mHealth can be effective in improving outcomes such as increasing patient adherence and healthy behaviors in chronic disease management [9], and reducing ED use [10-12]. According to 2 systematic reviews of cost effectiveness of digital interventions (eg, telephone, SMS text messaging, and mHealth apps), for those studies that included some type of economic evaluation, there is some evidence that these interventions may be cost-beneficial [9,13]. Even in the mHealth interventions where the outcomes were not statistically significant or cost-savings were not addressed, a reduction in preventable and nonemergent ED visits suggest likely significant cost-savings for health care payers in the community [13].

Given that mobile phones can reach traditionally hard to reach groups, including people experiencing homelessness [14], mHealth interventions should be considered when designing interventions to decrease ED and hospital visits in people experiencing homelessness. In a small pre-post study, people experiencing homelessness indicated that it was easy to use a mobile phone to participate in an mHealth intervention, and a qualitative study of people experiencing homelessness in the pre-post study found that mobile phones empowered people experiencing homelessness to self-manage their health and social needs [15,16]. A scoping review of 12 studies found that using technology on mobile phones such as SMS text messaging and apps could help improve access to mental health services for youth experiencing homelessness, with evidence of feasibility and acceptability by users [17]. The scalability of mHealth interventions for people experiencing homelessness will depend, in part, on whether its benefits outweigh the costs of the intervention. Thus, in this study, we will estimate the costs of an mHealth intervention, which will be described in the Methods section, that was implemented to decrease ED and hospital use among people experiencing homelessness and conduct an exploratory financial cost-benefit analysis and preliminary budget impact analysis to evaluate the potential cost-savings from this intervention for people experiencing homelessness.

## Methods

### Overview

Interactive Care Coordination and Navigation (iCAN) is a community-based mHealth intervention in the United States that was designed using findings from previous studies related to acceptability and benefits of mHealth interventions in people experiencing homelessness [15,16] and with feedback from homeless care providers and people with the lived experience of homelessness. The purpose of iCAN, which is implemented at community-based sites that provide homeless services, is to improve care coordination between health and social services for people experiencing homelessness with a goal of reducing hospitalizations and ED visits. It is a multicomponent intervention comprised of SMS text messages, preinstalled apps and resources, including a bus pass, and a case manager who conducted telephone encounters and used bidirectional SMS texting to communicate with participants. Participants received 3‐5 messages daily regarding medication adherence and appointment reminders, general health messages, motivational messages such as encouragement to keep goals, and as needed messages for local information (eg, weather updates and resource fair information). Participants were also able to text the case manager or study staff. Within 72 hours of enrollment, the case manager conducted a telephone assessment to identify relevant health and social needs whereby they could connect participants to medical and social services in the community. Case managers received weekly reports twice about ED and hospital use from the local health information exchange (HIE) and contacted participants within 72 hours of an ED or hospital visit to help coordinate care needs for managing discharge instructions. People experiencing homelessness who were 18 years or older, owned a cell phone with service, had at least 2 chronic health conditions, had at least 2 hospitalizations or ED visits 6 months before enrollment, scored 4 or higher on the Rapid Estimate of Adult Literacy in Medicine-Short Form, and scored greater than 17 on the Mini-Mental State Exam were eligible to participate in iCAN. There was a total of 60 participants in the iCAN intervention. The mean age of participants in the iCAN intervention was 50.1 (SD 10.2) years. Most (42/60, 70%) were men; over half (34/60, 57%) reported their race as White and 32% (19/60) reported their race as Black. The randomized controlled trial (RCT) of iCAN completed recruitment in August 2023 and data analysis is ongoing.

Informed by previous studies of mHealth interventions [18-21], we conducted a three-part economic evaluation of iCAN, including a (1) cost analysis, (2) exploratory financial cost-benefit analysis, and (3) budget impact analysis. For the cost analysis, we used expenditure data from the iCAN RCT to determine the total cost and cost per participant per year (PPPY). Using these costs, we conducted an exploratory cost-benefit analysis to understand the minimum health care savings that would cause iCAN to have a positive return on investment. Finally, we conducted a budget impact analysis to determine the financial requirements of scaling up the iCAN intervention in a metropolitan area. For the cost and cost-benefit analyses, we used scenario and probabilistic sensitivity analyses to determine the effect of different assumptions and uncertainty in existing data on our findings.

We followed the Consolidated Health Economic Evaluation Reporting Standards (CHEERS) [22], the International Training and Education Center for Health (I-TECH) Applied Economic Evaluation of Digital Health Interventions Report [23], the The International Society for Pharmacoeconomics and Outcomes Research (ISPOR) Budget Impact Analysis Report [24], and other guidance [25] in conducting this study. We used a public-payer perspective, assuming that the majority of people experiencing homelessness do not have private health insurance and cannot pay for out-of-pocket health care costs [26,27]. Thus, the costs of iCAN and health care services used by people experiencing homelessness are borne by the public sector through public health care payers (eg, Medicaid), health care systems, and local governments.

### Data Collection

#### Intervention Costs

We collected expenditure records from the ongoing iCAN RCT to determine intervention costs. Intervention costs were separated into startup and recurring costs using a microcosting approach [28].

Startup or capital costs were separated into participant-related and program-related costs. Participant-related startup costs included a cell phone, phone case, armband, charging cable and block, rechargeable power bank, and drawstring backpack. Program-related startup costs included the initial payment for the SMS text messaging platform and printing costs for training materials.

Recurring costs were separated into participant-related, program-related, and personnel costs. Participant-related recurring costs included a monthly bus pass and a SIM card with a phone plan, which are given to each participant. Program-related recurring costs included monthly SMS text messaging platform maintenance fees and HIE data access fees. Access to HIE data are required to monitor ED and inpatient health care use of participants during the trial, however, this expense may be excluded during the scaling up of iCAN in the community. We explored this scenario in sensitivity analysis.

Personnel-related recurring costs included the salaries of a case manager and program coordinator. The case manager spent approximately 20 hours per week communicating with 24 enrolled participants; for 60 participants, this was equivalent to 1.25 case managers. The program coordinator, a role assumed by graduate research assistants during the iCAN trial, is responsible for several operational and administrative tasks, including setting up participants’ cell phones (1 h per phone), training participants on the intervention (1 h per participant), and troubleshooting any technology-related issues (2 h per week). The case manager’s annual salary was estimated based on the average hourly wage of social workers in Texas from the Bureau of Labor Statistics multiplied by the total work hours (2500) per year [29]. The salary of the program coordinator was based on the average hourly pay of graduate research assistants, which is available on the human resources website of The University of Texas at Austin [30]. The hours spent by the program coordinator on specific tasks were determined through interviews with trial staff and a review of staff records, and the total time spent was used to calculate the program coordinator’s annual salary.

#### Health Care Costs

We used existing data to estimate the annual health care costs related to inpatient and ED care among people experiencing homelessness. We relied on previous studies on health care utilization among people experiencing homelessness in Texas to determine the average number and range of ED [31-34] and inpatient visits [31,35] (Multimedia Appendix 1). We took the weighted average and median of ED and inpatient visits across studies, which resulted in equivalent values and were comparable with the estimates from other jurisdictions and the United States [36-40]. We then multiplied these figures by the average cost of each service from the 2022 Medical Expenditure Panel Survey-Household Component (MEPS-HC) database [41]. With the average cost of inpatient (US $16,609, [SE $860]) and ED visits (US $1233 [SE $66]) from MEPS-HC, we used a total health care cost of US $36,916 PPPY. This average cost is comparable with what previous health care utilization studies among people experiencing homelessness who use health care services have reported in Texas and other locations [31-40].

### Analysis

#### Cost Analysis

We calculated the total costs of iCAN by adding up all the startup and recurring intervention costs. We also calculated the PPPY costs by dividing the total costs by 60, which is the number of enrolled participants in the iCAN RCT. In scenario analyses, we varied assumptions around the cost inputs (Multimedia Appendix 2) and calculated total and PPPY costs under each scenario.

#### Cost-Benefit Analysis

Using the PPPY costs of iCAN, we calculated the minimum savings or reduction in the average health care costs among people experiencing homelessness that would lead to a benefit-cost ratio >1 for iCAN, where the financial benefits associated with the intervention outweigh its costs. We calculated this “savings threshold” by dividing the PPPY cost of iCAN by the average health care costs among people experiencing homelessness multiplied by 100%. For example, if the PPPY cost of iCAN is 10% of the average health care costs among people experiencing homelessness, a health care cost reduction of >10% (the savings threshold) would lead to a benefit-cost ratio >1 for iCAN. These savings can be accrued by reducing the number or severity of ED and inpatient visits. We then calculated the benefit-cost ratio of iCAN under different savings thresholds from 0% (no savings) to 50%.

To account for uncertainty in various cost inputs (ie, cost of the iCAN, number of ED, and inpatient visits), we conducted a probabilistic sensitivity analysis. A common approach in economic modeling, probabilistic sensitivity analysis involves generating probability distributions for each input using the best available parameter values and then drawing 10,000 random values of each input to produce a distribution of results. From these 10,000 estimates, we determined the mean, median, and 95% probable interval of the savings threshold for iCAN. We assigned gamma distributions for iCAN and health care costs and Poisson distributions for the annual number of inpatient and ED visits among people experiencing homelessness.

#### Budget Impact Analysis

We conducted a budget impact analysis to determine the financial requirements of implementing iCAN for people experiencing homelessness in a metropolitan area. We used a 5-year time horizon and selected Travis County, Texas, which includes the city of Austin where the iCAN trial was conducted, as the setting of the analysis. The number of unsheltered people experiencing homelessness in the first year was assumed to be 3990 based on estimates of the Homelessness Response System [42]. We assumed that the people experiencing homelessness population would grow annually by 3.4%, which is the estimated population growth rate for people experiencing homelessness by the US Department of Housing and Urban Development in 2023 [43]. We further assumed that only 60% of people experiencing homelessness would be eligible to participate in iCAN based on previous use of ED and inpatient services based on trial data and previously published studies. Finally, we assumed that only 80% of eligible people experiencing homelessness would participate in iCAN based on engagement data from the trial.

We included fixed and variable startup costs and variable recurring costs in the budget impact analysis. Fixed start-up costs, which were only paid in the first year of program implementation regardless of the number of participants, included the costs of the SMS text messaging platform and iCAN training materials. Variable startup costs included the 1-time cost of supplies (eg, cell phone) per participant. Recurring costs were the cost of services and salaries that varied based on the number of participants; these costs included SMS text messaging platform maintenance fees, HIE data access, personnel salaries, and annual bus passes and phone plans for each participant. We used a medical inflation rate of 2.3% in estimating future costs.

### Ethical Considerations

The trial described in the study was approved by the University of Texas Institutional Board (STUDY00002666). The consent form indicated that research data would be kept for use in secondary analyses.

## Results

### Cost Analysis

The total cost of implementing iCAN to 60 participants for 1 year was US $170,921. The startup costs of iCAN (US $15,925) were estimated to be 9.3% ($15,925/$171,921) of the total cost (Table 1) and the remainder ($155,996/$171,921, 90.7%) were attributable to recurring costs (US $155,996), particularly personnel costs (US $84,908; Table 2). The startup and recurring costs PPPY were US $265 and US $2600, respectively, leading to a total PPPY cost of US $2865 PPPY (Table 3).

**Table 1.** Startup costs of Interactive Care Coordination and Navigation, a mobile health intervention for people experiencing homelessness in the United States, for a cost analysis. Total costs are calculated based on 60 participants. Costs are in 2022 US $.

Item		Unit cost per participant (US $)	Total cost (US $)
Participant-related			
	Cell phone	149.99	8999.40
	Phone case	12.99	779.40
	Arm band	8.99	539.40
	Charging block	3.50	210.00
	USB-C charging cable	3.50	210.00
	Power bank	14.99	899.40
	Drawstring backpack	1.34	80.40
	Subtotal	195.30	11,718.00
Program-related			
	Text messaging platform implementation	59.80	3588.00
	Printing costs for training binder supplies	10.31	618.59
	Subtotal	70.11	4206.59
Total		265.41	15,924.59

**Table 2.** Recurring costs of Interactive Care Coordination and Navigation, an mobile health intervention for people experiencing homelessness in the United States, for a cost analysis. Total costs were calculated for 60 participants. Costs are in 2022 US $.

Item		Unit cost (US $)	Units	Total annual cost (US $)
Participant-related				
	Bus pass	41.25 per month	12 months per participant	29,700
	Sim card or phone plan	210 per 6 months	12 months per participant	25,200
	Subtotal	—^a^	—	54,900
Program-related				
	SMS text messaging platform maintenance	99 per month	12 months for all participants	1188
	HIE^b^ data access	200 per hour	750 hours for all participants	15,000
	Subtotal	—	—	16,188
Personnel-related				
	Program coordinator^c^	17 per hour	224 hours for all participants	3808
	Case worker	32.44 per hour	2500 hours for all participants	81,100
	Subtotal	—	—	84,908
Total		—	—	155,996

a Not applicable.

b HIE: health information exchange.

c Includes time spent on setting up (1 h/participant) and training (1 h/participant) participants and maintenance (2 h/wk).

**Table 3.** Per participant per year costs of Interactive Care Coordination and Navigation, a mobile health intervention for people experiencing homelessness in the United States, for a cost analysis. Costs are in 2022 US $.

Category		Cost PPPY^a^ (US $)
Startup cost		
	Participant-related	195.30
	Program-related	70.11
	Subtotal	265.41
Recurring cost		
	Participant-related	915.00
	Program-related	269.80
	Personnel-related	1415.13
	Subtotal	2599.93
Total cost		2865.34

a PPPY: per participant per year.

The total PPPY cost of iCAN intervention varied slightly under different scenarios (Table 4). For example, when startup costs were excluded (Scenario A), recurring costs PPPY was US $2600. Excluding the cost of HIE data access (Scenario C) led to a similar PPPY cost of US $2615. The removal of bus passes and HIE data access (Scenario D) produced the lowest cost of US $2120 PPPY.

**Table 4.** Results of scenario analyses for a cost analysis of Interactive Care Coordination and Navigation, an mobile health intervention for people experiencing homelessness in the United States. All costs are in PPPY^a^ 2022 US $.

Type of cost	Base case	Scenario A	Scenario B	Scenario C	Scenario D
Startup costs (US $)	265	0	265	265	265
Recurring costs (US $)	2600	2600	2105	2350	1855
Total iCAN^b^ costs (US $)	2865	2600	2370	2615	2120
Savings threshold	7.8	7.0	6.4	7.1	5.7

a PPPY: per participant per year.

b iCAN: Interactive Care Coordination and Navigation.

In Table 4, the savings threshold refers to the percent (%) reduction in average health care costs for ED and inpatient care among people experiencing homelessness. The base case includes both startup and recurring costs. Scenario A includes recurring costs only. Scenario B includes startup costs and recurring costs without the bus pass. Scenario C include startup costs and recurring costs without HIE data access. Scenario D includes startup costs and recurring costs without the bus pass and HIE data access.

### Cost-Benefit Analysis

**Figure 1.**
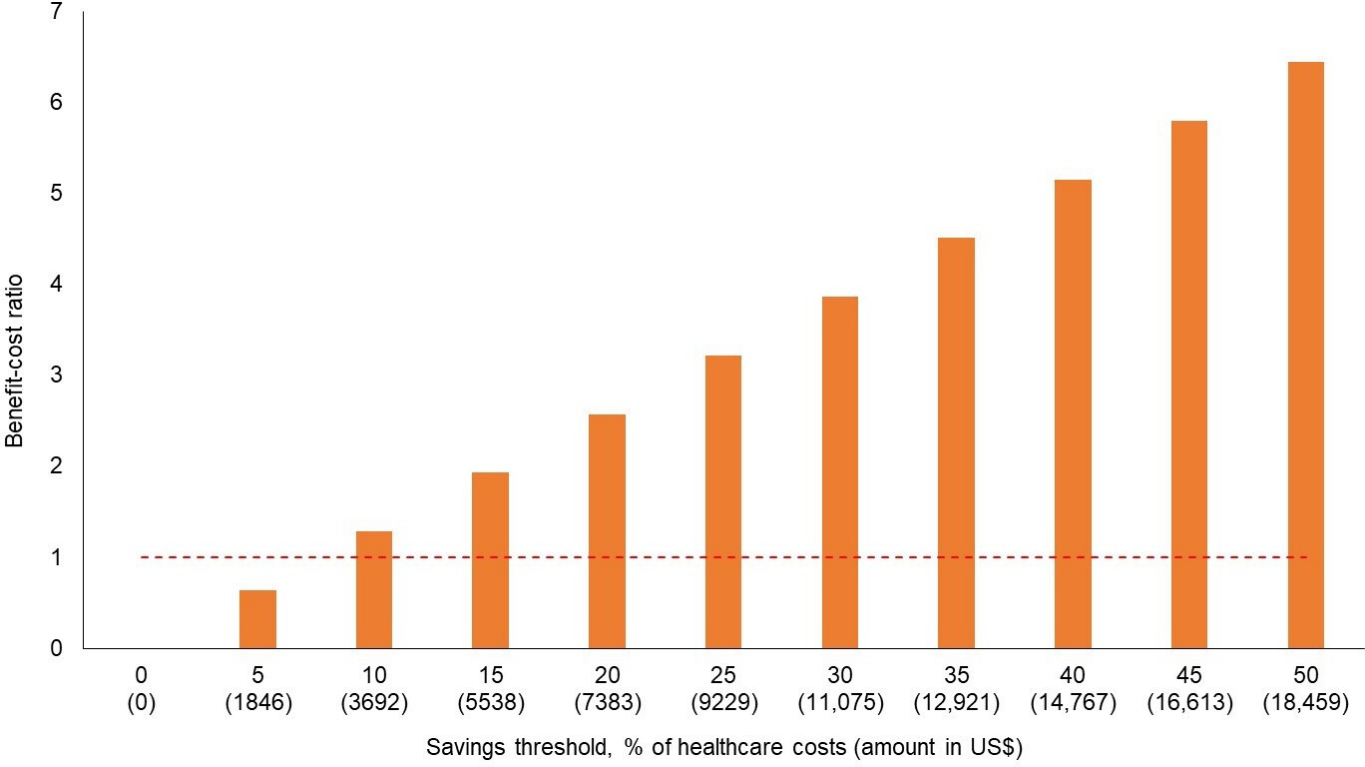
Benefit-cost ratio of Interactive Care Coordination and Navigation, an mobile health intervention for people experiencing homelessness in the United States, under varying savings thresholds.

When the cost of iCAN PPPY equals the base-case estimate (US $2865), the savings threshold is 7.8% (Table 2). This means that if average health care costs (US $36,917) among people experiencing homelessness were reduced by more than 7.8% through iCAN, the financial benefits would outweigh the costs of the intervention. A higher savings threshold is associated with a higher benefit-cost ratio for iCAN (Figure 1). When 10% ($3692/$N36,917) of average health care costs among people experiencing homelessness are averted (equal to the average cost of 3 ED visits), iCAN has a benefit-cost ratio of 1.29. When health care costs are reduced by 25% ($9229/N$36,917; equal to 56% [$9229/N$16,609] of the average cost of an inpatient visit), the benefit-cost ratio is 3.22, which means that iCAN produces US $2.22 in health care savings per US $1 spent.

Figure 1 shows the benefit-cost ratio of iCAN under different savings thresholds, which is the percent (%) reduction in average health care costs for ED and inpatient care among people experiencing homelessness. Average health care costs were calculated using the weighted average of ED and inpatient visits among people experiencing homelessness from previous studies, whose value was equivalent to the median. The dotted red line marks a benefit-cost ratio of 1, which occurs when the benefits or financial savings of iCAN outweigh its costs. The probabilistic sensitivity analyses, which account for uncertainty in the costs of iCAN and the average health care costs of people experiencing homelessness, found a median savings threshold of 8.2% (95% probable interval 3.2‐94.0). The average savings threshold was higher at 21%.

### Budget Impact Analysis

The 5-year financial cost of implementing the iCAN intervention in Travis County for 10,250 people experiencing homelessness was around US $28.7 million (Table 5). If at least 5% of average health care costs among people experiencing homelessness are averted, the net budget impact is US $9.7 million. If average health care savings increased to 7%, the net budget impact of iCAN is further reduced to US $2.2 million.

**Table 5.** A 5-year budget impact analysis of Interactive Care Coordination and Navigation, an mobile health intervention for people experiencing homelessness in the United States (the 5-year total costs were calculated for the total number of people experiencing homelessness participating in Interactive Care Coordination and Navigation and health care savings were calculated based on the average health care costs for emergency department and inpatient care among people experiencing homelessness per year [US $36,917] among those who use these services).

		Year 1	Year 2	Year 3	Year 4	Year 5	5-year total
Total number of participants, n		1915	1980	2048	2117	2189	10,250
Intervention costs (US $)							
	Fixed costs	4207	0	0	0	0	4207
	Startup costs, PPPY^a^	195	195	195	195	195	2,001,764
	Recurring costs, PPPY	2600	2600	2600	2600	2600	26,648,502
Total financial cost (US $)		5,357,637	5,535,448	5,723,653	5,918,257	6,119,478	28,654,472
Net budget impact (US $)							
	5% health care savings	1,822,466	1,880,080	1,944,002	2,010,099	2,078,442	9,735,088
	7% health care savings	408,397	417,933	432,142	446,835	462,028	2,167,335

a PPPY: per participant per year.

## Discussion

### Principal Results

In this study, we found, using a base-case estimate of US $2865, that a reduction in ED and hospital costs (estimated to be US $36,916 PPPY) of about 8%, which equals the cost of 2 ED visits PPPY, through iCAN would outweigh the costs of the intervention. Higher reductions in ED and hospital costs would result in a higher benefit-cost ratio for iCAN. While the economic value of mHealth interventions have been previously explored, none have focused on interventions for people experiencing homelessness. In total, 29 out of 39 studies evaluated in a systematic review conducted by Iribarren et al [44] in 2017 concluded that mHealth interventions show cost-effectiveness, economic benefit, or cost savings. Just over half of the mHealth interventions included SMS text messaging as the mHealth-related function in the intervention, with 77% (17/22) of these interventions reporting positive cost outcomes [44]. However, many limitations were identified in this systematic review. Of the 39 studies included in the review, only 12 were considered high-quality economic evaluations, which was defined as meeting at least 90% (21/24) of the items in the CHEERS checklist. In 2022, Gentili et al [13] conducted a systematic review of digital health interventions, which includes mHealth and other technologies such as telehealth, evaluating studies published after the time period evaluated by Iribarren et al [44]. Furthermore, 35 studies were included, and the findings were relatively consistent with that of Iribarren et al [44]. Only 12 of the 35 studies met at least 90% (21/24) of the items on the CHEERS checklist [13]. While the quality of economic evaluation was the focus of these systematic reviews, there is little evaluation of the budgetary impact and scalability of the included studies. Budget impact analyses were not conducted for these studies, and any evaluations on the economic scalability and sustainability of the interventions were preliminary, suggesting the need for formal budget impact analyses on mHealth interventions.

Another mHealth intervention used SMS to increase participation in their own care and decrease the number of no-show appointments in homeless veterans at a metropolitan Veterans Health Administration hospital. There was a significant decrease in the number of appointments cancelled by the patient and no-show appointments, and there was a significant reduction in ED visits. This study also showed that there were significant cost-savings when comparing the costs of implementing and maintaining SMS appointment reminders with the cost of unutilized services due to cancelled and no-show appointments [45]. A systematic review conducted in 2014 found that studies that evaluated cost-effectiveness showed cost savings associated with SMS services compared with standard of care while maintaining equal efficacy of interventions [46]. Even in the mHealth interventions where the outcomes were not statistically significant or cost-savings were not addressed, a reduction in preventable and nonemergent ED visits suggest likely significant cost-savings for health care payers in the community [13,44,47].

Our study adds to this literature by estimating the minimum health care savings needed for an mHealth intervention like iCAN to have positive net financial benefits. However, our analysis excludes other benefits that may be associated with iCAN, such as improvements in health outcomes and health-related quality of life. These health-related benefits should be quantified, valued, and added to the cost-benefit analysis or cost-effectiveness analysis to gain a more comprehensive picture of iCAN’s overall value.

Research has shown that people experiencing homelessness use more health care services than individuals with stable housing. In our analysis, we relied on previously published estimates of ED and inpatient health care use among people experiencing homelessness in Texas, which all found that use of these services is common (>1) among people experiencing homelessness who have a history of using these services. For example, single-center retrospective studies in Harris County, Texas, and Fort Worth, Texas found that people experiencing homelessness who used the ED had an average of 3.82 and 3.29 ED visits, respectively [33,34]. These numbers align with recent estimates from the National Hospital Ambulatory Medical Care Survey that found an ED visit rate of 310 per 100 persons per year in 2020‐2021[37]. Estimates of inpatient visits among people experiencing homelessness in Texas are more limited and appear higher than numbers from other jurisdictions. A study of veterans in Pittsburgh, Pennsylvania, estimated the average number of inpatient visits at 0.7 (SD 1.6) among those who were hospitalized in 2012‐2013, while single-center studies in Boston, Massachusetts, and Seattle, Washington State, found slightly higher numbers (>2) [40,48], which align with research showing a high risk of readmission among people experiencing homelessness [2,3]. Using these health care utilization numbers, we calculated an average health care cost comparable with previously published estimates of the cost of care among people experiencing homelessness [35,49,50]. Additional research is needed to understand the health care use and costs among people experiencing homelessness.

This study not only provides insight to the cost-benefit of the iCAN mHealth intervention, but also highlights the scalability and sustainability of an mHealth intervention like iCAN in a metropolitan area. Including a budget impact analysis of iCAN demonstrates that expanding the services provided by iCAN over a 5-year period is both possible and cost-saving. While existing literature on the economic implications of mHealth interventions exists, the majority of these studies did not conduct formal budget impact analyses to determine the sustainability of implementing an intervention on a larger scale [13,44]. Our economic evaluation and budget impact analysis balances both real-world data with necessary assumptions on a realistic scale, and many of the assumptions used in this study can be replaced with more specific real-world data after the iCAN RCT has concluded.

This economic evaluation and budget impact analysis provides insightful information in an emerging field where concrete data are lacking in several aspects. In published studies conducting budget impact analyses, the interventions being studied are typically medications or treatment protocols, not multicomponent behavioral health interventions [21,51-54]. While there are dozens of economic evaluations of mHealth interventions, there are very few formal budget impact analyses associated with these economic evaluations [13,44]. Our economic evaluation and budget impact analysis aims to fill this gap about mHealth interventions and the economic scalability and sustainability associated with expanding the services provided.

### Limitations

The primary limitations of this study involve the assumptions made in the economic evaluation. The costs associated with the iCAN intervention were estimated from expenditure data from the RCT, which likely overestimate the actual costs of the intervention if implemented in real-world setting due to costs required to ensure accurate trial design and adherence to protocol [20]. We addressed this issue by adjusting the fixed costs included in our budget impact analysis, however, other economies of scale are potentially still excluded from our analysis. Health care costs related to ED and inpatient visits were estimated using national health care expenditure data (MEPS-HC) because such data were not available through the trial or for Travis County specifically. With possible variations in health care cost and utilization among people experiencing homelessness, our probabilistic sensitivity analyses explored the impact of uncertainty on our findings and indeed found a wide range of savings thresholds for iCAN. This suggests iCAN’s economic value will be highly influenced by the change in health care costs and utilization of people experiencing homelessness who receive the intervention. Finally, we also had to make simplifying assumptions in the budget impact analysis, including the current size and growth of the people experiencing homelessness population in Travis County over the next 5 years. Our budget impact analysis can be easily updated with more precise inputs to understand the financial aspects of expanded access to iCAN.

### Conclusions

This exploratory economic evaluation gives estimates of the cost of the iCAN mHealth intervention while exploring the cost-savings and budget impact of implementing the iCAN mHealth intervention on a larger scale. By using data from the iCAN RCT and other available resources, this economic evaluation determined that implementing the iCAN mHealth intervention for people experiencing homelessness in a metropolitan area provides financial cost-benefit if 1 hospitalization or 2 ED visits can be avoided. Future studies should explore the feasibility of implementing the iCAN mHealth intervention using data from the completed iCAN RCT to limit uncertainty in the cost of scaling up the intervention. Health care payers should examine the benefits of implementing mHealth interventions like this one to prevent unnecessary health care utilization and the associated costs among people experiencing homelessness.

## Supplementary material

10.2196/64973 Multimedia Appendix 1 Annual emergency department and inpatient health care utilization among people experiencing homelessness.

10.2196/64973 Multimedia Appendix 2 Scenarios explored in the cost analysis of Interactive Care Coordination and Navigation, an mHealthmobile health intervention for people experiencing homelessness in the United States.

## Abbreviations

CHEERS: Consolidated Health Economic Evaluation Reporting Standards
ED: emergency department
HIE: health information exchange
I-TECH: International Training and Education Center for Health
iCAN: Interactive Care Coordination and Navigation
ISPOR: The International Society for Pharmacoeconomics and Outcomes Research
MEPS-HC: Medical Expenditure Panel Survey-Household Component
mHealth: mobile health
PPPY: per participant per year
RCT: randomized controlled trial
