# Supplementary material for: The Interactive Care Coordination and Navigation mHealth Intervention for People Experiencing Homelessness: Cost Analysis, Exploratory Financial Cost-Benefit Analysis, and Budget Impact Analysis
Source: JMIR Form Res. 2025 Mar 18;9:e64973. doi: 10.2196/64973 (PMC11936304; doi:10.2196/64973)
Supplement: Multimedia Appendix 1 [file formative-v9-e64973-s001.docx]

| Type of health care use | | Setting and sample size, location (n^a^) | Average number of visits per year^b^ | Weighted average^c^ | Median^c^ |
| --- | --- | --- | --- | --- | --- |
| **Emergency department** | |  |  | 3.0 | 3.0 |
|  | Amato et al [33] | Harris County, Texas (986) | 3.82 |  |  |
|  | Holmes et al [34] | Fort Worth, Texas (1613) | 3.29 |  |  |
|  | Petrovich et al [31] | Tarrant County, Texas (451) | 2.59 |  |  |
|  | Schiefelbein et al [32] | Austin, Texas (2401) | 3.30 |  |  |
| **Inpatient** | |  |  | 2.0 | 2.0 |
|  | Buck et al [35] | Harris County, Texas (331) | 3.02 |  |  |
|  | Petrovich [31] | Tarrant County, Texas (222) | 1.35 |  |  |

^a^Refers only to the number of people experiencing homelessness in each study.

^b^Among those who used this healthcare service.

^c^Rounded off to the nearest whole number.
