# Supplementary material for: The Interactive Care Coordination and Navigation mHealth Intervention for People Experiencing Homelessness: Cost Analysis, Exploratory Financial Cost-Benefit Analysis, and Budget Impact Analysis
Source: JMIR Form Res. 2025 Mar 18;9:e64973. doi: 10.2196/64973 (PMC11936304; doi:10.2196/64973)
Supplement: Multimedia Appendix 2 [file formative-v9-e64973-s002.docx]

| Scenario | Startup costs | | Recurring costs | | |
| --- | --- | --- | --- | --- | --- |
|  | Participant-related | Program-related | Participant-related | Program-related | Personnel |
|  |  |  |  |  |  |
| Base case | ✓ | ✓ | ✓ | ✓ | ✓ |
| Scenario A | —^a^ | — | ✓ | ✓ | ✓ |
| Scenario B | ✓ | ✓ | Excluding bus pass | ✓ | ✓ |
| Scenario C | ✓ | ✓ | ✓ | Excluding HIE^b^ | ✓ |
| Scenario D | ✓ | ✓ | Excluding bus pass | Excluding HIE | ✓ |

^a^Not applicable.

^b^HIE: health information exchange.
